# Supplementary material for: Heterologous expression of the cyanobacterial fructose-1,6−/sedoheptulose-1,7-bisphosphatase in Chlamydomonas reinhardtii causes increased cell size and biomass productivity in mixotrophic conditions
Source: Algal Res. Author manuscript; Available in PMC 2026 Jan 8. (PMC7618607; doi:10.1016/j.algal.2025.104208)
Supplement: Supplementary [file EMS211880-supplement-Supplementary.pdf]

Supplementary Figure 1. FBP/SBPase purification from *Chlamydomonas reinhardtii* expressing lines. Immunoblot against Strep-Tag II (A) and Coomassie blue stained gel (B) performed on the FBP/SBPase purified using the Strep-Tactin XT resin column. (C) Quantitative SDS-PAGE for quantification of the recombinant FBPase/SBPase using CrFBPase as a standard.

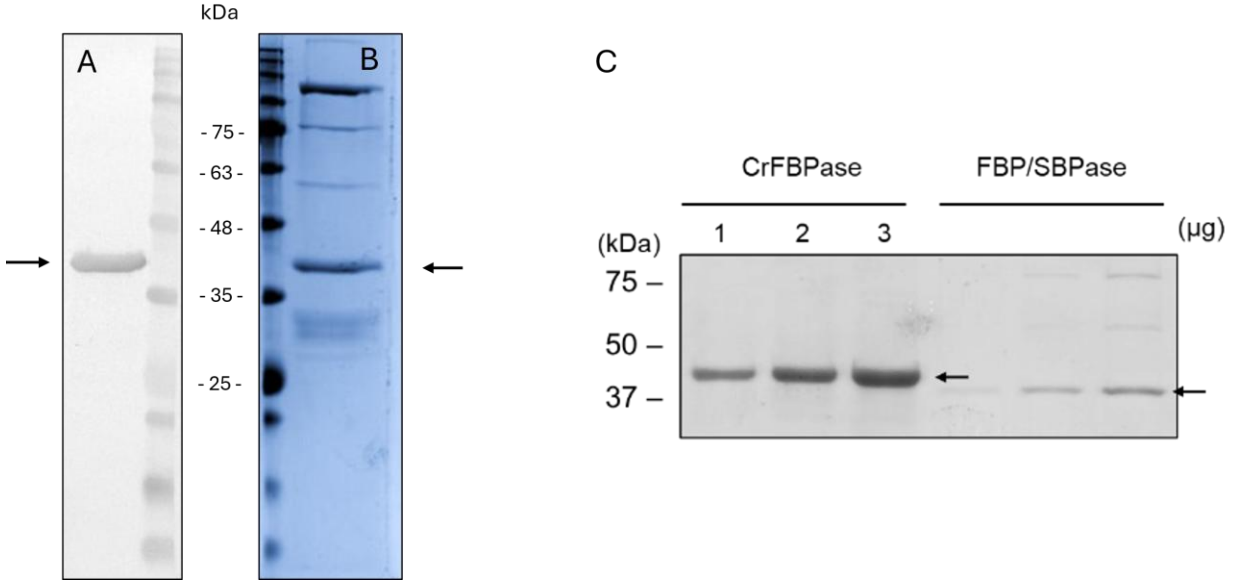

Supplementary Figure 2. *In vitro* analysis of FBPase catalytic activity of recombinant FBP/SBPase. A representative experiment of dose-dependent effect of fructose-1,6-phosphate (FBP) on the activity of purified FBP/SBPase from expressing lines is reported.

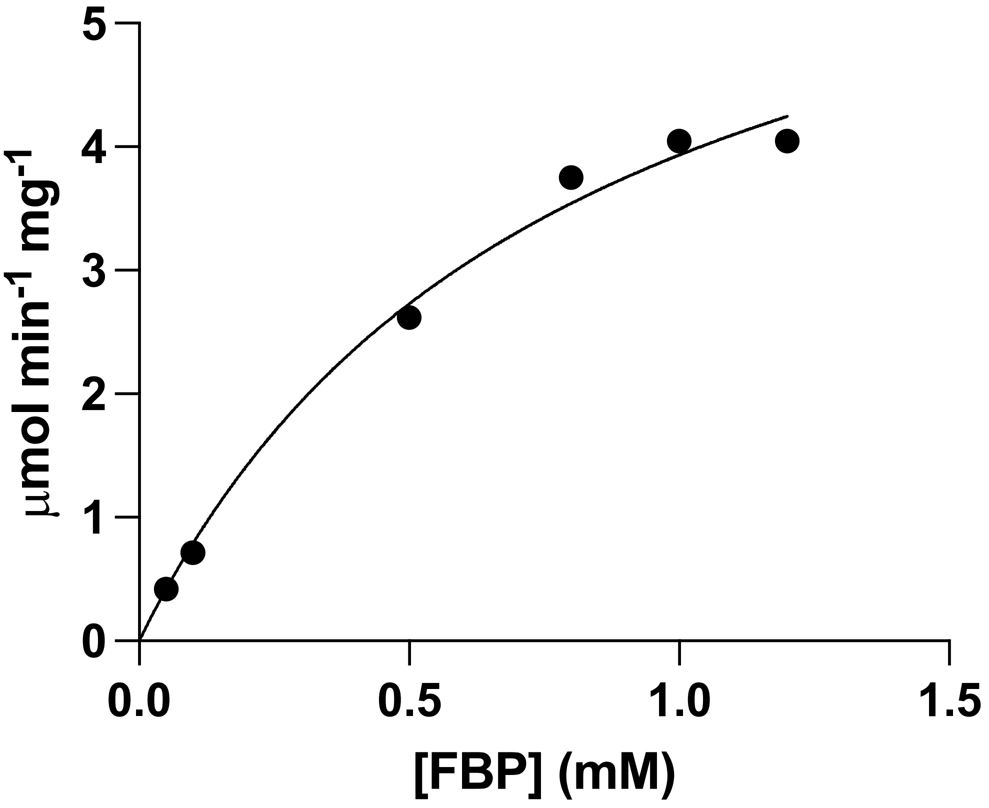

Supplementary Figure 3. Gene expression of cyanobacterial FBP/SBPase and endogenous FBPases and SBPase. Gene expression of cytosolic FBPase, SBPase, chloroplast FBPase, FBP/SBPase, and the house keeping rack1 was evaluated by semi quantitative RT-PCR. Relative gene expression with respect to UVM4 was estimated by densitometry on images of DNA agarose gel migration.

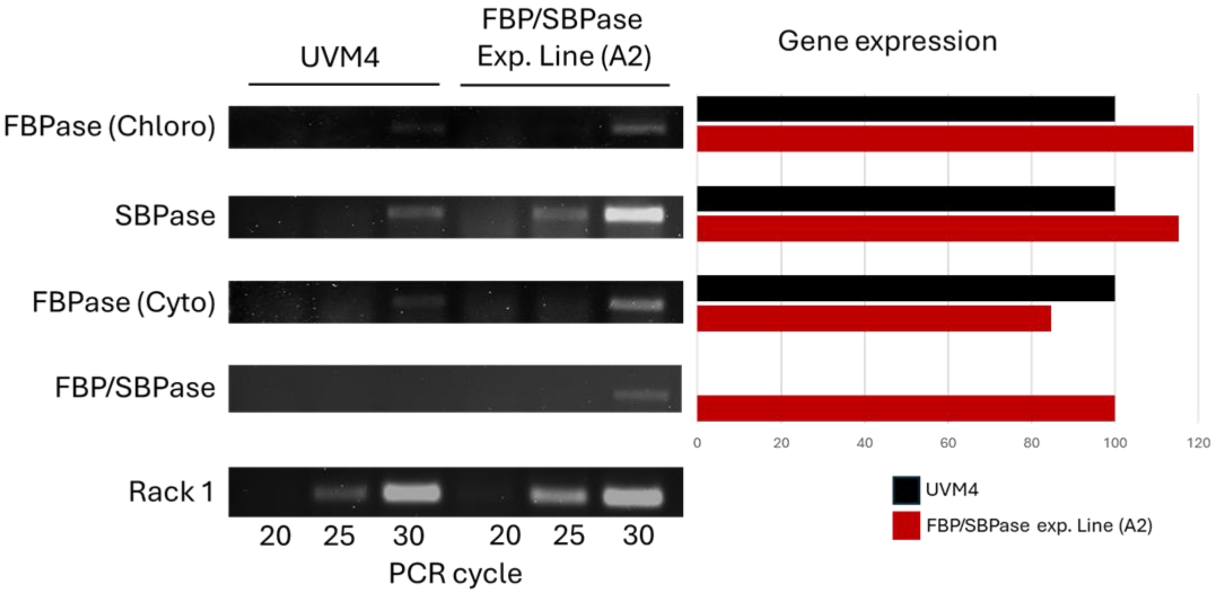

Supplementary Figure 4. Chlorophyll content per cell in FBP/SBPase expressing lines compared to the UVM4 background Chlorophyll content on cell basis in UVM4 (black bar) and expressing lines (red bar).

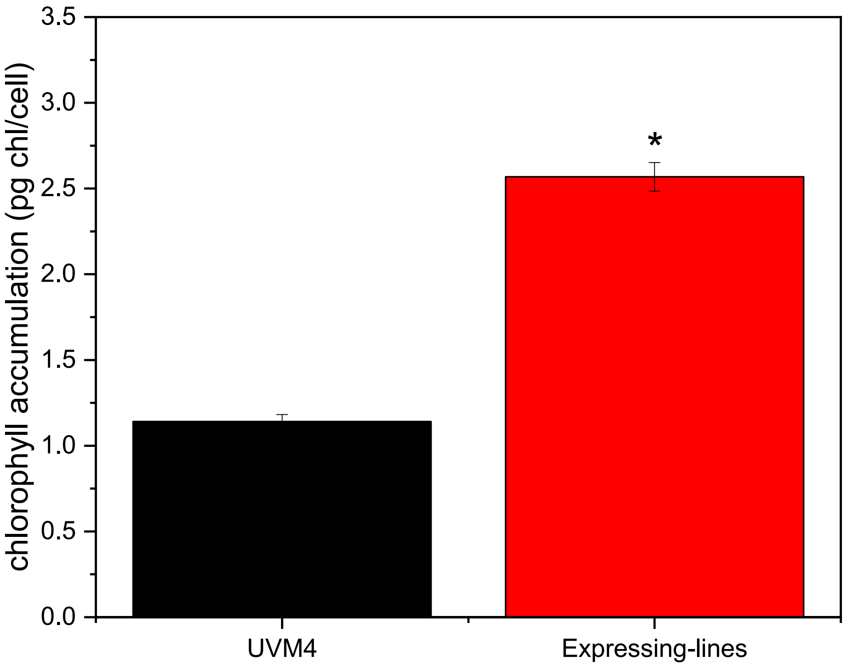

Supplementary Figure 5. Impact of FBP/SBPase expression on Photosystem II photochemical and non-photochemical activity. (A) PSII operating quantum yield (Y(II)), (B) non-photochemical quenching (NPQ) measured at different actinic lights in dark-adapted cells, (C) PSII electron transport rate (ETR(II)), (D) redox state of plastoquinone (1-qL). Data reported are means of three biological replicates with standard deviation shown.

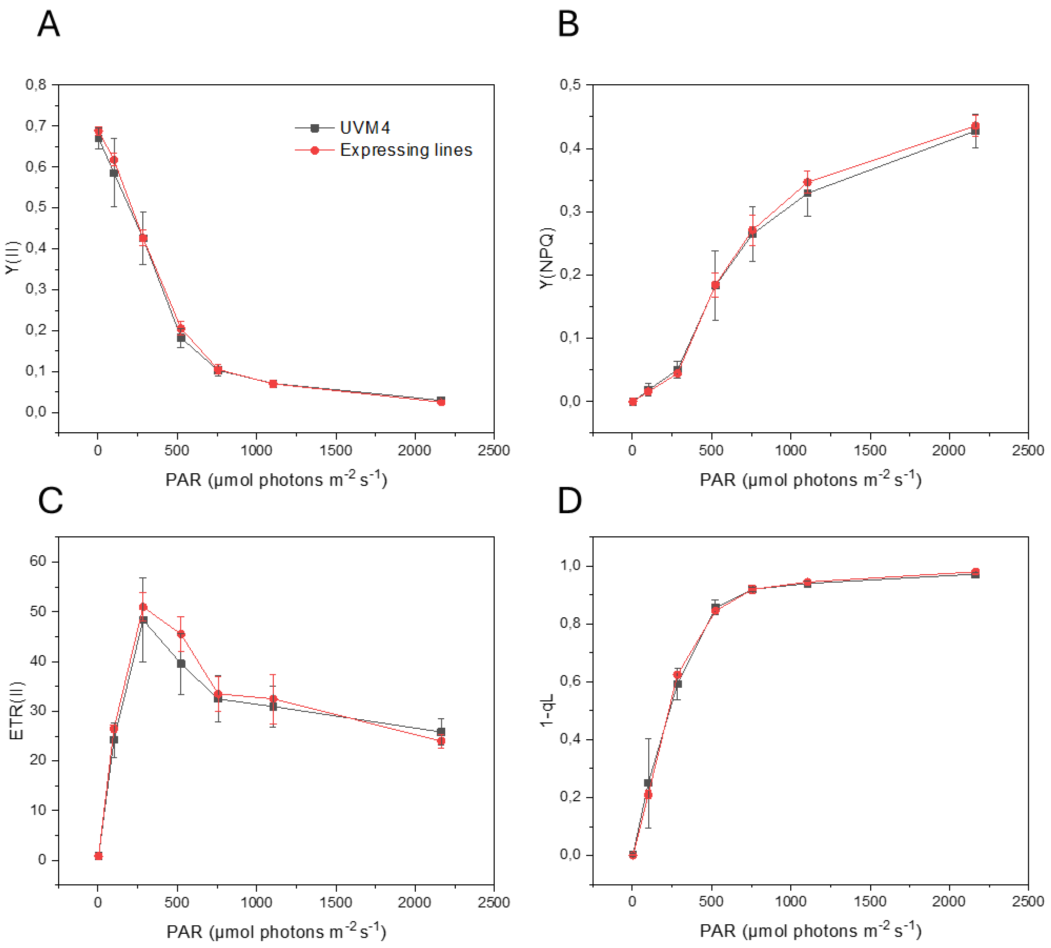

Supplementary Figure 6. Biomass productivity of UVM4 and FBP/SBPase expressing lines. Growth curves of UVM4 (black) and expressing lines (red) cultivated at 100 µmol photons m^{-2} s^{-1} in photoautotrophy, monitoring OD at 720 nm (A). Volumetric biomass (B), cell density (C) and cellular diameter (D). Error bars are reported as standard deviations ($n = 4$). Values significantly different compared to UVM4 are reported with * (Student's t test, $P < 0.05$)

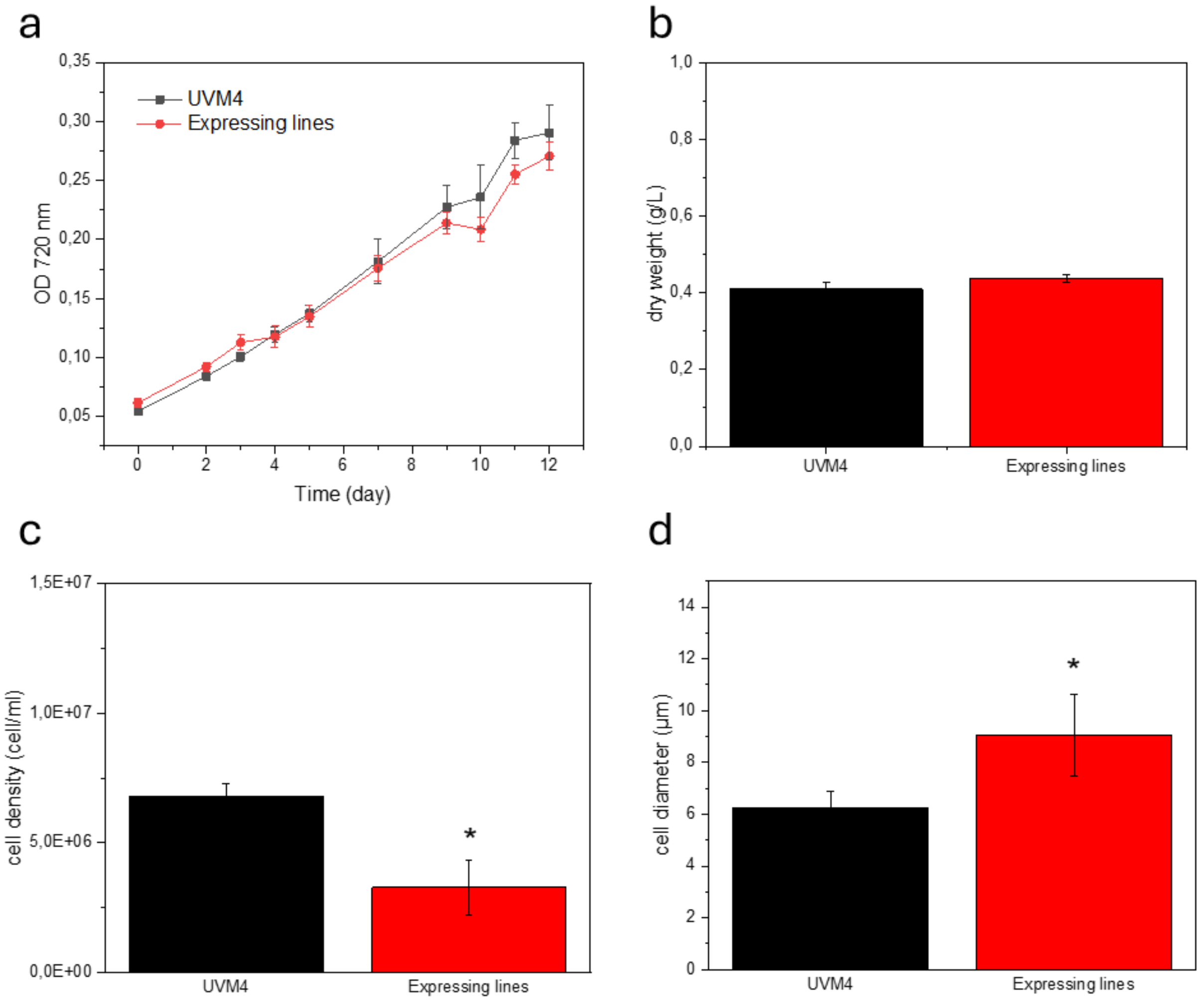

Supplementary Figure 7. Morphology of FBP/SBPase expressing cells. Microscopy images of background and engineered cells. Images of UVM4 (left) and FBP/SBPase expressing lines (A2 and B9, right) grown in TAP medium at exponential phase are shown with scale bar (5 μm).

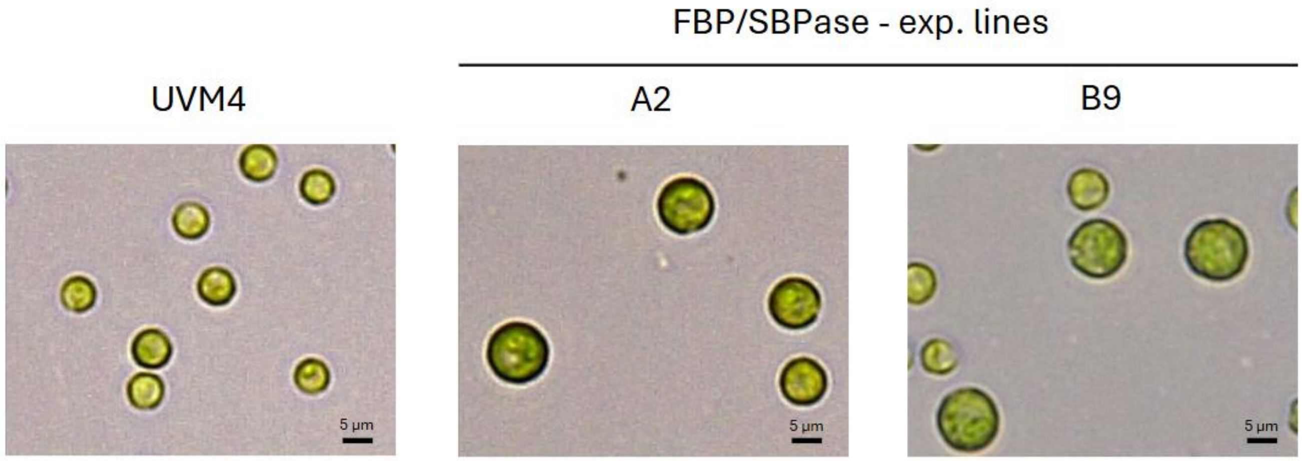

Supplementary Figure 8. Cell density and cell volume of UVM4 and FBP/SBPase expressing lines in mixotrophy conditions in low light at different time points. (A) Growth curves of UVM4 (black) and expressing lines (red) cultivated at 100 µmol photons m^{-2} s^{-1} in mixotrophy, monitoring cell density (cell mL^{-1}). (B) Cell volume at different time points. Values significantly different compared to UVM4 are reported with * (Student's t test, $P < 0.05$). (C) The culture volume determined by multiplying the cell density (A) by the cell volume (B). Error bars are reported as standard deviations (n = 4).

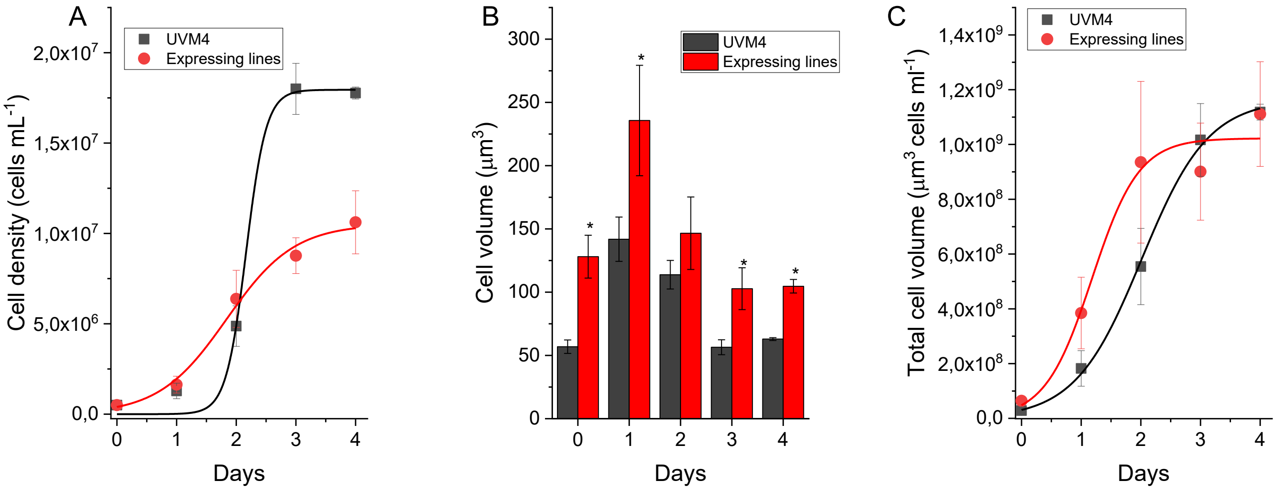

Supplementary Figure 9. Effect of FBP/SBPase expression on algal growth in mixotrophy. Growth curve generated by following cell scattering at 720 nm and volumetric productivity for UVM4 (black) and FBP/SBPase expressing lines (red). Two different light intensities were used: 100 µmol photons m^{-2} s^{-1} for low light (LL) and 1000 µmol photons m^{-2} s^{-1} for high light (HL) with air with atmospheric or 3% enriched CO_2 concentration. Error bars are reported as standard deviations (n = 4).

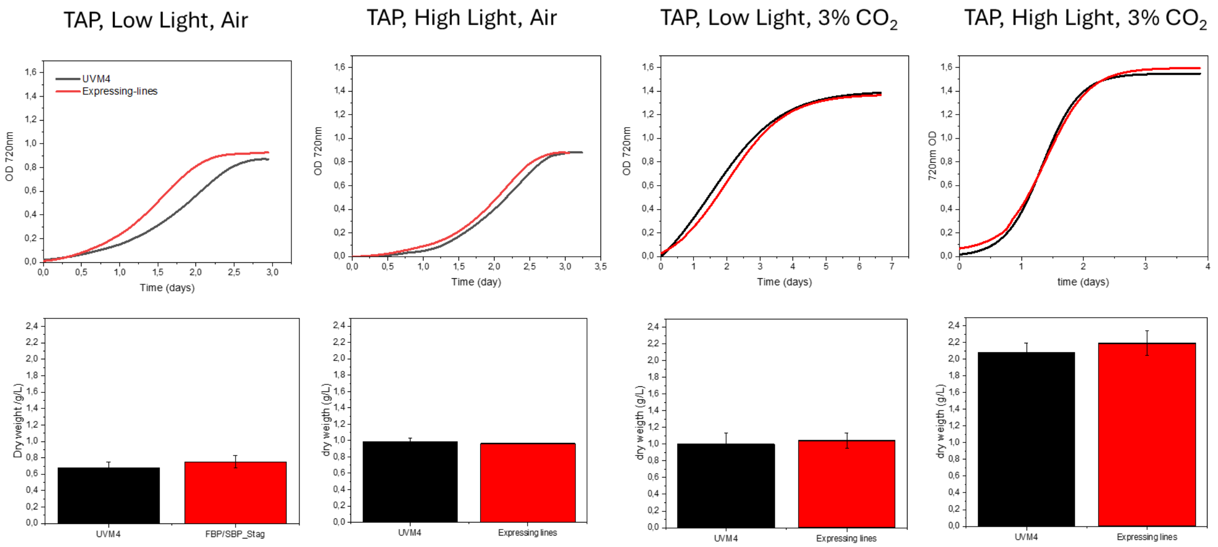

Supplementary Figure 10. Effect of FBP/SBPase expression on cell density and dimension. Cell density (A) and cellular volume (B) of UVM4 (black) and FBP/SBPase expressing lines (red) at the end of growth reported in Supplementary Figure 7. Error bars are reported as standard deviations (n = 4). Values significantly different compared to UVM4 are reported with * (Student's t test, $P < 0.05$)

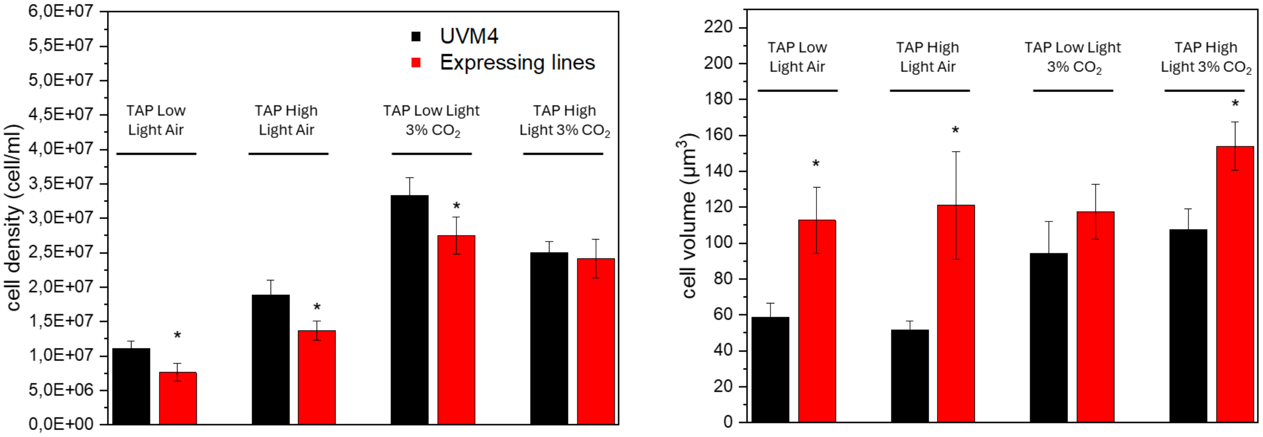

Supplementary Table 1. Fitting results of oxygen evolution curves. Net oxygen evolution data were obtained upon subtraction of oxygen consumption rate in the dark and fitted with the hyperbolic function y Pmax * x/(KI + x), where Pmax is the maximum net oxygen evolution rate and KI the light intensity at which the net oxygen evolution rate is Pmax/2. Values significantly different compared to UVM4 are reported with * (Student's t test, $P < 0.05$)

		UVM4			EXPRESSING LINES		
TAP	Pmax (µmolO2/cell*h)	9.01e-08	±	1.27e-09	2.21e-07 *	±	3.36e-09
	Pmax (µmolO2/rg Chl*h)	7.90e-08	±	1.12e-09	8.63e-08	±	1.31e-09
	KI (µmol m^{-2} s^{-1})	118.4	±	9.9	208.7 *	±	15.7
HS	Pmax (µmolO2/cell*h)	2.12e-07	±	7.04e-09	3.31e-07*	±	1.46e-08
	Pmax (µmolO2/rg Chl*h)	1.95e-07	±	9.47e-09	1.51e-07	±	8.82e-09
	KI (µmol m^{-2} s^{-1})	225.3	±	27.2	215.6	±	35.3
